# Efficacy and tolerability of a low-dose, 2-week administration of sunitinib followed by a week rest (2/1 schedule) for metastatic renal cell carcinoma: a single center experience of six cases

**DOI:** 10.1186/1756-0500-7-872

**Published:** 2014-12-04

**Authors:** Katsuhiro Makino, Kenji Yoda, Junzo Tomoishi, Haruki Kume

**Affiliations:** Department of Urology, Ome Municipal General Hospital, 4-16-5 Higashiome, Ome, Tokyo, Japan; Department of Urology, The University of Tokyo, Tokyo, Japan

**Keywords:** Sunitinib, Metastatic renal cell carcinoma, Renal cancer medical treatment, Treatment toxicity, Tyrosine kinase inhibitors

## Abstract

**Background:**

Sunitinib, an oral multitarget tyrosine kinase inhibitor and standard first-line treatment for metastatic renal cell carcinoma (mRCC), is generally administered on a 6-week schedule (4 weeks on/2 weeks off). However, drug toxicity often leads to temporary treatment interruption, resulting in reduced treatment efficacy. In this report, we investigated whether sunitinib administration of at a dose of 25 mg/day in a 2-weeks-on/1-week-off cycle would reduce the incidence of drug-related side effects while maintaining drug efficacy.

**Findings:**

A total of six patients with mRCC were orally administered sunitinib at a dose of 25 mg/day in a 2-weeks-on/1-week-off regimen until intolerable toxicities occurred. All enrolled patients were assessed for toxicity and response. The median treatment period was 24 months (range, 9–40 months). Objective responses were as follows: disease stabilization of >6 months was achieved in all patients. The most important toxicities were neutropenia, fatigue, and proteinuria, but all were controlled.

**Conclusions:**

Oral sunitinib at 25 mg/day in a 2-weeks-on/1-week-off regimen to Japanese patients can avoid drug-related toxicities while achieving the same dose intensity as a 6-week schedule. Because these data were derived from a small number of patients, future prospective studies of modified sunitinib administration schedules are warranted.

## Background

Until a few years ago, metastatic renal cell carcinoma (mRCC) was considered a radio- and chemotherapy-resistant disease. The 5-year mRCC survival rate was estimated at <10% [1] because mRCC is highly resistant to conventional chemotherapy, which involves the use of cytotoxic drugs. Therefore, only a small proportion of patients (approximately 20%) has benefited from the use of cytokine therapy with interferon-α or interleukin-2, while obtaining a mean survival of 10–12 months [2–4]. Recently introduced molecularly targeted agents, such as those with activities against the vascular endothelial growth factor (VEGF) or platelet-derived growth factor (PDGF) receptor pathways, present promising novel treatment modalities to inhibit angiogenesis, leading to the hypervascularization observed in mRCC [5, 6]. Of these molecularly targeted drugs, sunitinib has been observed to be the most promising for the treatment of mRCC. Sunitinib is a multi-target tyrosine kinase inhibitor specific to VEGF and PDGF receptors and can also effectively block the actions of other tyrosine kinases [7]. At present, standard treatment for mRCC involves oral sunitinib at 50 mg/day in 4/2-week cycles. However, this agent has also been associated with intolerable adverse events necessitating dose reduction or temporary treatment discontinuation, although tyrosine kinase inhibitors require continuous administration until disease progression. Therefore, prolonged exposure even to low-grade toxicity presents major discomfort to patients, resulting in a deterioration of quality of life [8, 9]. One of the most important requirement for successful targeted molecular therapy is to continue treatment until disease progression; therefore, special attention must be paid to monitor drug-induced side effects. Importantly, an adequate dose intensity of the drug must be maintained to prevent tumor growth during the sunitinib off-dose periods.

The aim of the present study was to investigate whether oral sunitinib administration at 25 mg/day in 3-week cycles of 2-weeks-on/1-week-off (2/1-week regimen) can reduce the incidence of the most significant drug-related side effects, as graded by criteria of the World Health Organization (WHO), while maintaining the standard planned drug dose intensity. A second endpoint was to verify the effectiveness of the 2/1-week regimen in terms of overall response (OR), where OR = complete response (CR) + partial response (PR), according to the Response Evaluation Criteria in Solid Tumors (RECIST).

## Findings

### Administration schedule and dosage of sunitinib

We evaluated the response of 6 Japanese patients with mRCC to oral sunitinib at 25 mg in a 2/1-week schedule. Each patient received an initial dose of sunitinib at 37.5 mg orally in a 4/2-week regimen. However, 5 patients developed grade 3 thrombocytopenia and grade 2 fatigue, and 1 patient developed febrile neutropenia within a month. Then, the dosage was reduced to 25 mg for four patients in a 4/2-week regimen, one patient in a 3/1-week regimen, and one patient in a 2/1-week regimen. If a patient wished to change the treatment schedule because of adverse events, the patient received the same dose of 25 mg/day in a 2/1-week regimen.

### Baseline evaluation

At the start of the administration of sunitinib and every 3–6 months thereafter, all patients underwent diagnostic monitoring of the tumor [chest, abdominal, and pelvic computed tomography scans, evaluation of Eastern Cooperative Oncology Group (ECOG) performance status; and laboratory testing of blood chemistry and urine]. Thyroid function was also periodically evaluated by monitoring serum concentrations of thyroid-stimulating hormone (TSH), triiodothyronine, and thyroxine. Treatment efficacy was determined using RECIST, and adverse events were evaluated by Common Terminology Criteria for Adverse Events v4.0.

### Patient characteristics

A total of six patients with mRCC (median age, 66 years; age range, 56–79 years) treated at Ome Municipal General Hospital (Tokyo, Japan) from July 2010 to October 2013, and written informed consent were obtained from the patients. Patient clinical characteristics are presented in Table 1. At the time of joining the study, all patients presented with an ECOG performance status of 0 or 1 and metastatic disease, of which 3 patients had metastatic disease in two or more sites, with the lung as the most frequently affected organ. All patients underwent total nephrectomy and were cleared of renal cell carcinoma. Memorial Sloan–Kettering Cancer Center risk classification was favorable in three cases, and intermediate in other cases. Sunitinib was administered to 5 patients as a first-line treatment, whereas the remaining patient had previously received cytokine treatment. The median treatment duration was 24 months (range, 9–40 months).

**Table 1:** **Patient characteristics**

No.	Age	Gender	BSA (m ^2^)	MSKCC risk	Metastatic sites	Prior therapy
1	70	Female	1.298	Intermediate	Lung, Lymph nodes	None
2	58	Female	1.446	Favorable	Abdominal wall, Lung, liver	None
3	56	Male	1.929	Intermediate	Lung	Interferon
4	79	Male	1.710	Favorable	Pancreas, Contralateral kidney	None
5	62	Male	1.893	Favorable	Pleura	None
6	64	Male	1.659	Intermediate	Lung	None

BSA, body surface area; MSKCC, Memorial Sloan-Kettering Cancer Center classification.

### Efficacy

The OR (OR = CR + PR), progression-free survival (PFS) of all six patients are summarized in Table 2. None of the patients achieved a CR for metastatic lesions in the lungs or lymph glands, although 5 patients attained a PR with an OR of 87%, and stable disease was achieved in the remaining patient. All responses were related to the target lesions. At the time of data analysis, all patients were alive and continued with therapy with this drug. The median PFS at this landmark time was 18 months (range, 6-40 months).

**Table 2:** **Treatment schedule and dosage of sunitinib**, **and efficacy**

No.	Dosage	Schedule	Duration of use	Response	PFS
1	37.5 mg	4 weeks on/2 weeks off	1 month	PR	19 months
	25 mg	4 weeks on/2 weeks off	2 months		
	25 mg	2 weeks on/1 week off	21 months		
2	37.5 mg	4 weeks on/2 weeks off	1 month	SD	9 months
	25 mg	4 weeks on/2 weeks off	2 months		
	25 mg	2 weeks on/1 week off	6 months		
3	37.5 mg	4 weeks on/2 weeks off	1 month	PR	17 months
	25 mg	2 weeks on/1 week off	26 months		
4	37.5 mg	4 weeks on/2 weeks off	1 month	PR	13 months
	25 mg	4 weeks on/2 weeks off	3 months		
	25 mg	2 weeks on/1 week off	9 months		
5	37.5 mg	4 weeks on/2 weeks off	1 month	PR	38 months
	25 mg	3 weeks on/1 week off	22 months		
	25 mg	2 weeks on/1 week off	17 months		
6	37.5 mg	4 weeks on/2 weeks off	1 month	PR	23 months
	25 mg	4 weeks on/2 weeks off	5 months		
	25 mg	2 weeks on/1 week off	18 months		

PR, partial response; SD, stable disease.

### Safety

Adverse events on 4/2 schedule and 2/1 schedule are summarized in Table 3. The most important sunitinib-related toxic effect was neutropenia (WHO grade ≥2), which was recorded in 2 patient. The incidence of fatigue (WHO grade ≥2) was more frequent, reported in 4 patients on 4/2 schedule, and 2/1 schedule decreased the grade in all cases except for two. TSH levels significantly increased in half of the patients, although it was not accompanied by clinical manifestations of hypothyroidism. Almost all patients (except for one) insisted on the 2/1-week regimen to reduce adverse events because it was easier to continue treatment than with the 4/2-week regimen.

**Table 3:** **Adverse events before and after schedule modification**

No.	25 mg, 4/2 schedule		25 mg, 2/1 schedule	
	Grade 2	Grade 3	Grade 2	Grade 3
1	Fatigue, emesis, leukopenia, neutropenia	None	Emesis, neutropenia	None
2	Fatigue, anorexia, dysgeusia, neutropenia, hand-foot syndrome	None	Fatigue, anorexia, dysgeusia, hand-foot syndrome	None
3	-	-	Neutropenia	None
4	Fatigue, dysgeusia	Proteinuria	Diarrhea	Proteinuria
5^†^	Neutropenia	None	None	None
6	Anorexia	Fatigue, mucositis, neutropenia	Fatigue	Neutropenia

^†^25 mg of sunitinib was administrated on 3/1schedule, instead of 4/2 schedule.

## Conclusions

Toxicity is often related to the activation of anti-angiogenic mechanisms. For example, among related side-effects, the swiftness of arterial hypertension onset is likely determined by the reduction of the microvascular bed with a consequent increase in peripheral resistance, and it is commonly observed that sunitinib-related hypertension tends to spontaneously revert to the normal range during sunitinib suspension [10]. Therefore, oncologists must manage the dose and administration schedule of sunitinib to maintain the dose intensity and simultaneously relieve side-effects.

Various sunitinib schedules have been evaluated for mRCC treatment, including a 3-week cycle comprised of treatment for 2 weeks followed by a 1-week rest period (schedule 2/1), a 4-week cycle of treatment for 2 weeks followed by a 2-week rest period (schedule 2/2), and a 6-week cycle of treatment for 4 weeks followed by a 2-week rest period (schedule 4/2). The findings of previous phase 1 trials recommend oral sunitinib at a dose of 50 mg using the 4/2 schedule [11]. Other clinical trials have investigated the possibility of guaranteeing equivalent sunitinib activity and efficacy and manageable tolerability in long-term mRCC treatment at a dose of 37.5 mg in a once-daily continuous dosing regimen [12]. However, the efficacy and safety profiles in these studies were not superior to those observed when sunitinib was administered using a 4/2-week schedule.

In our cases, we initiated sunitinib therapy at an initial dose of 37.5 mg using the 4/2 schedule because 50 mg may be too large a dose for Japanese patients, who tend to weigh less than Westerners. However, 37.5 mg still produced side-effects and prevented some patients from subsequent treatment; therefore, the dose was reduced to 25 mg in a 4/2-week cycle. Nonetheless, our patients continued to complain of toxicities such as fatigue, hand–foot syndrome, and mucositis, with the sunitinib accumulation after administration for 3 or 4 weeks. A direct relationship has also been suggested between toxicity and exposure to sunitinib; thus, we changed the schedule when patients wished to give up taking sunitinib because of severe adverse events, considering that the 2/1-week cycle would reduce the incidence of drug-related toxicity while maintaining the same efficacy due to the same dose intensity as the standard 4/2-week schedule. Response to sunitinib among our patient cohort, as marked by disease stabilization and extended PFS periods, appeared to closely resemble that of previous studies that enrolled greater patient numbers. Strikingly, the incidence of side-effects was lower than that reported for the 4/2-week schedule, and the 2/1-week schedule had a higher satisfaction level among patients. Based on our experience, the 2/1-week schedule presents a feasible treatment option that may lead to better tolerability and lower toxicity, while maintaining the required dose intensity. A shorter treatment cycle may result in a better toxicity profile with a reduced incidence of adverse events associated with longer drug exposure. However, these data were derived from a small number of patients; therefore, future prospective studies of modified sunitinib administration schedules are warranted.

In summary, oral administration of sunitinib at 25 mg for 2 weeks on 1-week off regimen to Japanese patients can avoid the occurrence of drug-related toxicity to achieve disease stabilization. A good treatment option would be to consider adaptation of cases.

## Abbreviations

mRCC: Metastatic renal cell carcinoma.
